# The current situation of treatment for patients suffering from schizophrenia in the Austrian forensic system

**DOI:** 10.1017/S1092852924000567

**Published:** 2025-03-24

**Authors:** Alexander Dvorak, Patrick Swoboda, Thomas Stompe

**Affiliations:** FTZ Göllersdorf, Wien, Austria

**Keywords:** Austrian forensic system, treatment of schizophrenia, involuntary treatment, preventive detention, not guilty for reasons of insanity

## Abstract

Treatment of patients suffering from schizophrenia in Austria: Treatment of patients with schizophrenia in the healthcare system is generally voluntary. This applies both to outpatient care provided by specialists in private practice, hospital outpatient clinics, or social psychiatric outpatient clinics and to inpatient care in hospitals. However, there is an exceptional situation in which the patient’s freedom of will is restricted by law. This is the case when acute danger to self or others caused by the disorder is present. With the involvement of the district court, the patient advocate, a possible adult representative, and an external expert, the patient’s freedom of movement can be restricted for a certain period of time to enable treatment. The acceptance of psychopharmacological therapy remains the patient’s decision in this situation, with the exception of explicit authorization by the court. Treatment under the consideration of proportionality, meaning that coercion is only applied in the case of an acute risk of severe bodily harm, is therefore possible for the majority of patients with schizophrenia. However, this does not mean that patients are able to connect to the care network in all cases. Some patients fail because the contact threshold is still too high. In order to reduce this, outreach care has been integrated into the existing services in many cases. These multi-professional teams often manage to establish contact with the patients and thus create a willingness to undergo treatment in order to counteract the long-term consequences, including complete social isolation and disintegration.

## Introduction

### Treatment of patients suffering from schizophrenia in Austria

Treatment of patients with schizophrenia in the healthcare system is generally voluntary. This applies both to outpatient care provided by specialists in private practice, hospital outpatient clinics, or social psychiatric outpatient clinics and to inpatient care in hospitals. However, there is an exceptional situation in which the patient’s freedom of will is restricted by law. This is the case when acute danger to self or others caused by the disorder is present. With the involvement of the district court, the patient advocate, a possible adult representative, and an external expert, the patient’s freedom of movement can be restricted for a certain period of time to enable treatment. The acceptance of psychopharmacological therapy remains the patient’s decision in this situation, with the exception of explicit authorization by the court. Treatment under the consideration of proportionality, meaning that coercion is only applied in the case of an acute risk of severe bodily harm, is therefore possible for the majority of patients with schizophrenia.

However, this does not mean that patients are able to connect to the care network in all cases. Some patients fail because the contact threshold is still too high. In order to reduce this, outreach care has been integrated into the existing services in many cases. These multi-professional teams often manage to establish contact with the patients and thus create a willingness to undergo treatment in order to counteract the long-term consequences, including complete social isolation and disintegration.

### Increase in patients with schizophrenia in the forensic system

As in many other European countries, the number of mentally ill patients in prison is increasing. In Austria, the number of inmates placed in forensic institutions has doubled in the last 20 years. This is due to both the rising number of admissions and the fact that releases have not kept pace with this increase. As far as admissions are concerned, there is a trend among people with schizophrenia, to name just 1 example, toward a shift in offense severity toward comparatively less serious offenses. During care in an inpatient forensic psychiatric setting, delays can occur due to limited therapeutic resources. Finally, in many cases, the search for a suitable outpatient aftercare facility once again proves to be a bottleneck. In order to take this development into account, an amendment to the law, the Measures Enforcement Adjustment Act, was passed in 2022. The plan was to ease the burden on the Austrian penitentiary system and improve legal certainty for mentally ill offenders who had committed relatively minor offenses such as resistance to state authority or dangerous threats.

## Legal situation to date

This legal package regulates the admission of mentally ill or disturbed criminals who are not guilty for reasons of insanity. The prerequisite for this is incapacity according to Section 11 of the Austrian Criminal Code (StGB), defined as follows:

Section 11 StGB: *Any person who, at the time of the offence, is incapable of understanding the injustice of his deeds or of acting in accordance with this understanding because of mental illness, mental disability, a profound disturbance of consciousness, or because of another serious mental disorder equivalent to one of these conditions, is not culpable.*

## Preventive detention for mentally ill offenders who are not culpable

If the person is incapable of guilt, it was previously sufficient for him/her to have committed an offense punishable by more than 1 year and a negative criminal prognosis to be committed to detention in accordance with Section 21 (1) StGB.

Around 75% of mentally ill offenders suffer from a schizophrenic disorder, 15% from an intellectual disability and 10% from an acquired organic brain disorder. Approximately half of them are housed in the 3 institutions belonging to the justice system (Forensic Therapeutic Centers), the rest in closed forensic wards in regional psychiatric hospitals. The resulting costs are reimbursed to the facilities by the Ministry of Justice.

If, after the arrest, there are sufficient grounds to assume that the requirements of Section 21 (1) StGB are met, the public prosecutor’s office must file an application for placement in an institution for mentally disturbed offenders.

If the offender’s mental state improves during provisional detention prior to the main hearing to such an extent that no further serious offense is to be feared, the court may refrain from unconditional committal.

Patients provisionally detained are also treated primarily in judicial departments of prisons.

If a person is unconditionally admitted to the preventive measure, the following legal requirements for conditional release apply:

*The purpose of placement in an institution for mentally disordered offenders is to prevent those placed there from committing punishable acts under the influence of their mental or emotional abnormality. The placement is intended to improve the condition of the inmates to such an extent that they can no longer be expected to commit punishable acts and to help the inmates to adopt a law-abiding attitude to life that is adapted to the requirements of community life.*

Duration of preventive *measures* associated with deprivation of liberty:

*Preventive measures shall be ordered for an indefinite period. They must be enforced for as long as their purpose requires. The court shall decide whether to end the preventive measure. Whether placement in an institution for mentally disturbed offenders is necessary shall be reviewed by the court ex officio at least once a year.*

**Figure:**
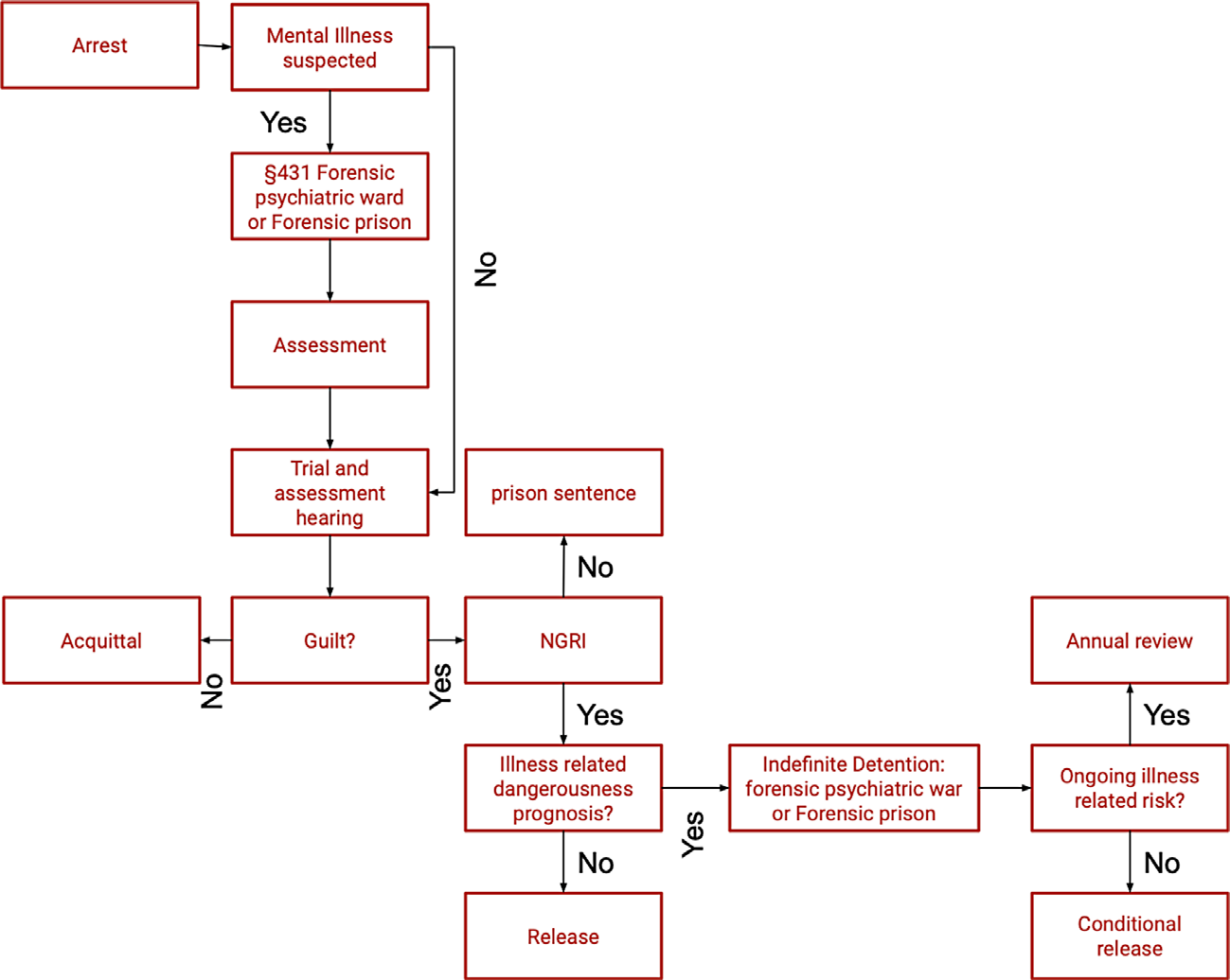

## Release from a preventive measure involving deprivation of liberty

Release from a preventive measure involving deprivation of liberty shall be ordered if it can be assumed from the performance and development of the detainee in the institution, his/her person, his/her state of health, his/her previous life, and his/her prospects for an honest future that the dangerousness against which the preventive measure is *directed no longer exists.*

The length of stay in detention thus depends on the reduction of the disease-specific dangerousness that led to the admission offense. In principle, it is not limited in time. Release is always subject to conditions.

The Ministry of Justice is responsible for the financing and logistics of the Austrian penitentiary system. The previous legal regulations led to 2 problems, which the legislator wanted to solve with the new Act of 2022:

## Increase in the prevalence of inmates in correctional facilities

**Figure 1.**
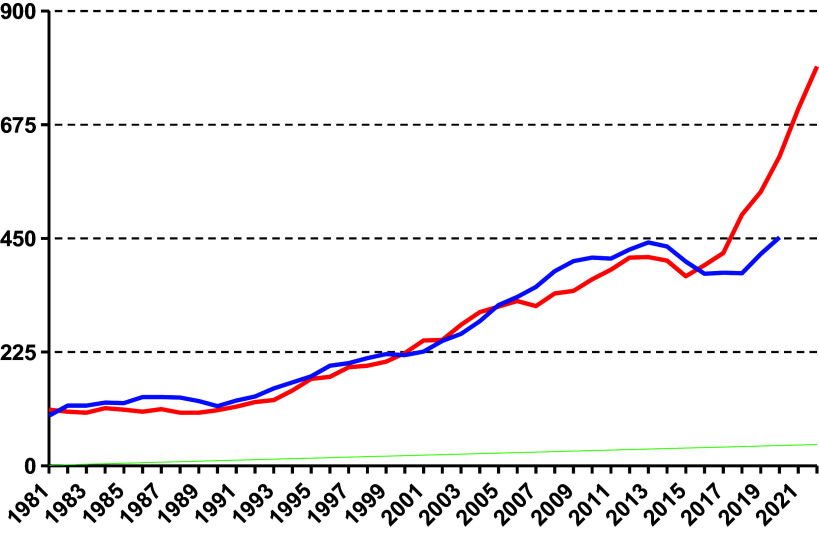
Prevalence from 1981 to 2022 of those placed in detention in accordance with Section 21 (1) (red) and (2) (blue) StGB.

Since 1980, the prevalence of offenders considered not guilty for reasons of insanity has risen continuously. Since 2015, the prevalence of offenders incapacitated for measures doubled to almost 800 inmates between 2014 and 2022 (Figure 1).

**Figure 2.**
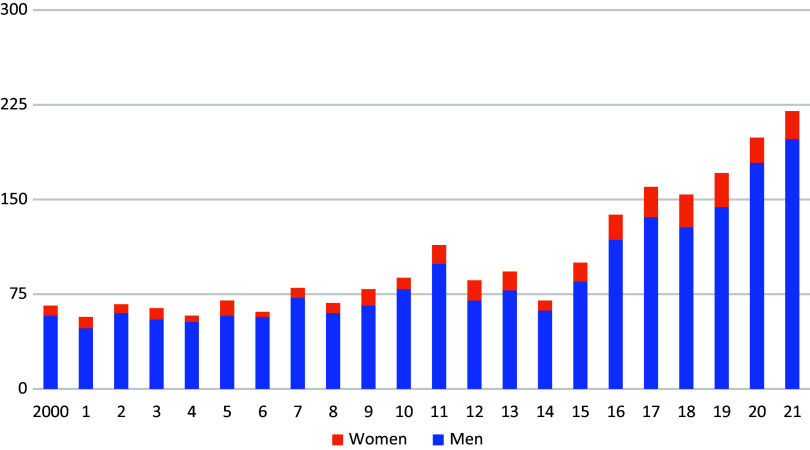
Admissions to detention in accordance with Section 21 (1) StGB by year, broken down by women and men (according to Ref. 1).

**Figure 3.**
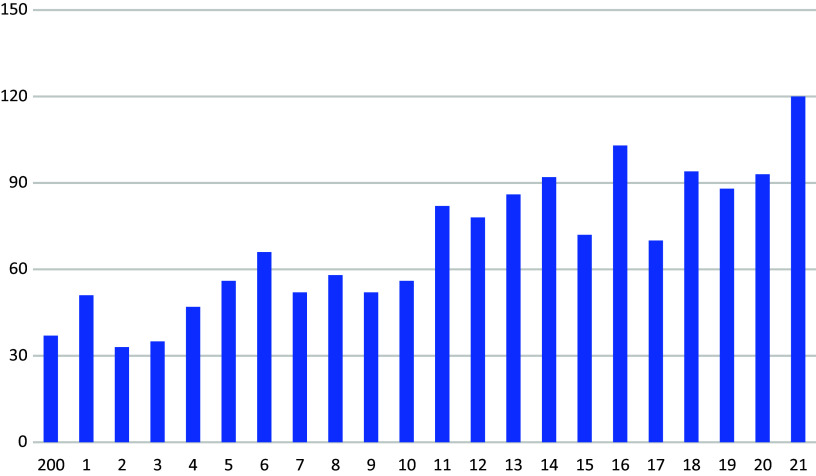
Conditional releases from detention under Section 21 (1) of the Criminal Code (according to Ref. 1).

With only a few exceptions, the number of annual admissions clearly exceeded the number of discharges (Figures 2 and 3).

**Figure 4.**
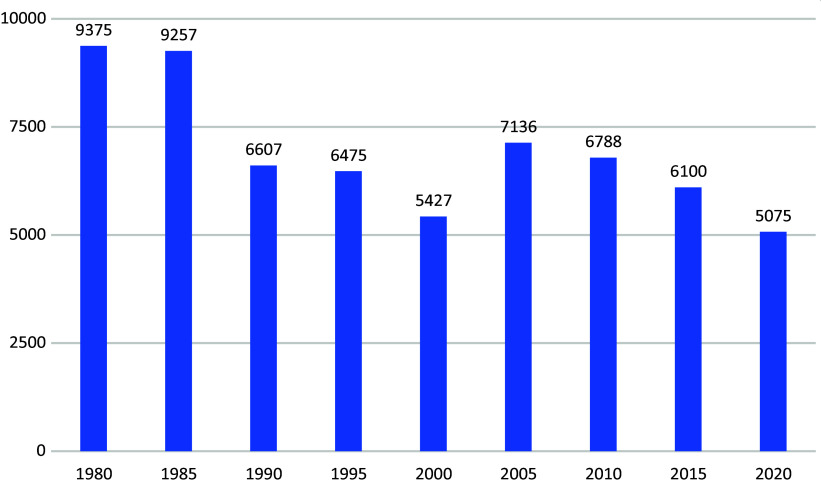
Final convictions for unconditional prison sentences 1980–2020 (Statistics Austria 2020—Crime statistics).

Most recently, 220 people were admitted to the penitentiary system in accordance with Section 21 (1) of the Criminal Code, compared to only 120 inmates who were released in the same year. However, the significant increase in admissions to the penitentiary system cannot be explained by a general increase in crime. Between 1980 and 2020, the number of offenders sentenced to unconditional custodial sentences fell by almost half (Figure 4), while the incidence of admissions to detention under Section 21 (1) quadrupled.

**Figure 5.**
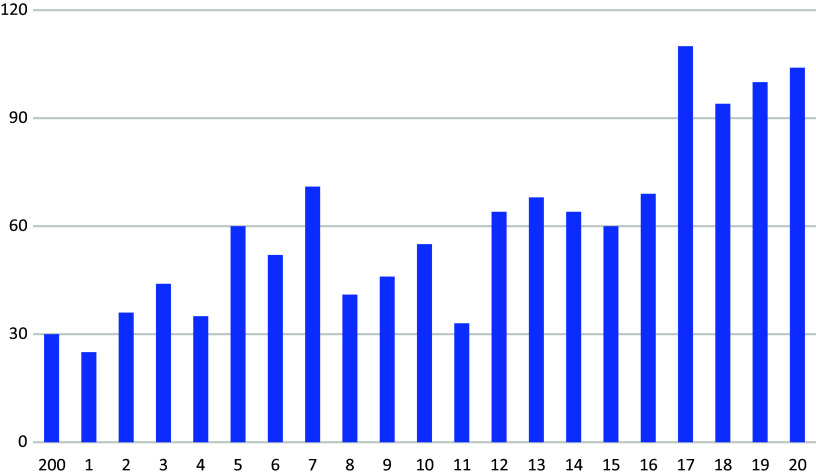
Provisional detentions under Section 429 (4) of the Code of Criminal Procedure by year, without subsequent committal to detention under Section 21 (1) of the Criminal Code (according to Ref. 1).

Provisional detentions of mentally ill offenders who were not ultimately committed to detention, also increased continuously from 2000 to 2020 (Figure 5).

### Reasons for the increase in forensic patients

If we look at the increase in the number of patients being cared for as part of forensic detention, it is clear that there is no monocausal explanation for this. When attempting to classify these reasons, a distinction can be made between pre-offense and post-offense treatment.

Before the offense, the changed conditions in general psychiatric care come to mind first. Without being able to break down the causes in detail, there was a reduction in available beds without outpatient care being able to compensate for this change. This is linked to a simultaneous reduction in admission times, also in order to have the necessary beds available for crisis interventions. However, this development also meant that the reasons for discharge from inpatient treatment changed. For example, patients who escaped the hospital were not readmitted or were discharged prematurely for disciplinary offenses such as illicit substance use or socially inadequate and aggressive behavior toward patients or staff.

In the category of post-offense reasons, at least 3 points should be mentioned here.

On the one hand, psychiatric experts are consulted by the court, perhaps even in the case of minor suspicions, with the result that patients who would not have been recognized as such in the past are now committed to preventive detention.

More far-reaching, however, are 2 points that influence the length of time patients spend in detention. The first point is that the personnel resources for treatment have not been able to keep pace with the increase in patients. This can delay the assessment of the relevance of the case and thus the start of treatment and extend the overall duration of treatment. Even if treatment in inpatient detention has been successfully completed, there is still 1 key point that needs to be clarified before discharge. And this key area is outpatient psychiatric and psychotherapeutic aftercare as well as a suitable place to live.

In addition to these reasons, a significant increase in the number of migrants admitted has been particularly noticeable in recent years (Stompe and Keckeis, 2017). In recent years, the proportion of inmates with a migration background has already exceeded the 50% mark.

All in all, multiple factors and participants play a role, which is why the number of patients in forensics continues to increase despite the commitment of treatment providers.

The increase in the prevalence of sane mentally abnormal offenders, which has, however, reached a plateau since 2014, probably has other causes. In the execution of measures in accordance with Section 21 (1), it is mainly personality-disordered and/or paraphilic offenders who are treated for offenses against sexual self-determination, primarily child sexual abuse and rape. The increase in prevalence is most likely due to the increasingly critical attitude of the population toward sexual offenses.

## Violation of the principle of proportionality

In addition to the question of how to ensure adequate care for mentally ill or disturbed lawbreakers given the sharp rise in incidences of admission and the relatively moderate rise in incidences of discharge, the proportionality of measures involving deprivation of liberty was increasingly discussed in Austria, as in Germany.

**Figure 6.**
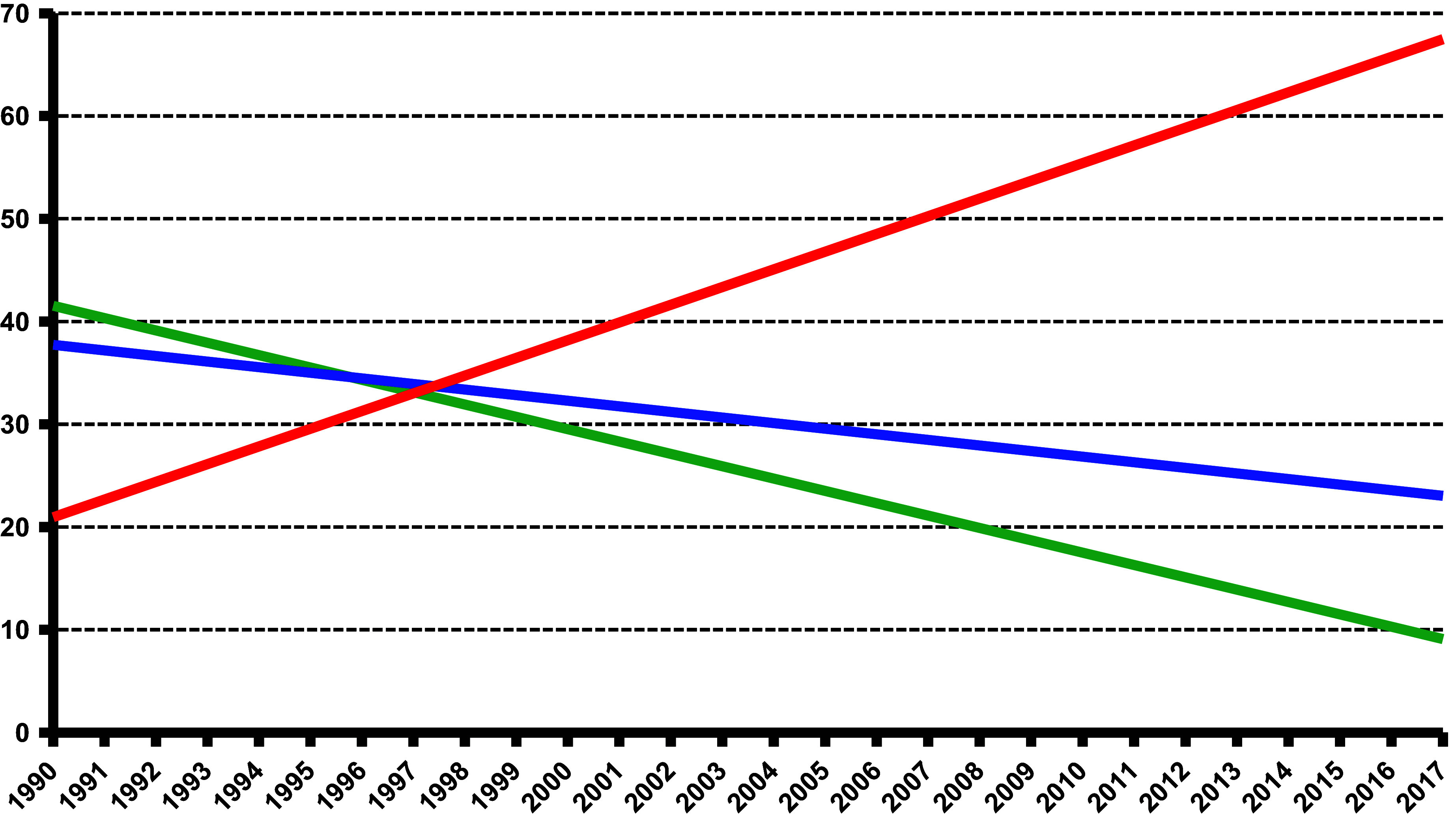
Change in the percentage shares of different offense types in the annual incidence of admissions to detention in accordance with Section 21 (1) StGB (1990–2017).

The available data show that the increases in the incidence and prevalence of imprisoned and the number of offenders imprisoned, particularly in correctional facilities in accordance with Section 21 (1) of the Criminal Code, are primarily attributable to persons who had committed relatively minor offenses such as dangerous threats or resistance to state authority, that is, offenses that are normally punishable by 1 year’s imprisonment (Figure 6).

Our research in the Forensic Therapeutic Center in Göllersdorf showed, that there is no correlation between the severity of the offense and the length of stay. This result is not entirely surprising, as treatment in the correctional facility is aimed at reducing the disease-specific dangerousness that led to the offense. The severity of the offense is not a criterion. The legislature clearly states that the dangerousness against which the preventive measure is directed, should no longer exist and that there should be prospects of a fair future. However, there are no indications that the severity of the offense for which the offender has been committed should be a criterion for the length of stay in preventive detention. Our research revealed that the type of illness also plays no role concerning how long a person is placed in a detention center. The decisive factors were an early age at the time of the first offense and an early onset and extent of the illness. Furthermore, patients with psychopathic personality traits were detained for longer, as they exhibited a higher degree of intramural deviant and aggressive behavior.

From the perspective of the principle of proportionality, the fact that mentally ill offenders who have committed less serious offenses such as resistance to state authority or dangerous threats, are treated for significantly longer in detention than is legally required for healthy offenders (Table 1). A comparison of the actual periods of imprisonment of healthy offenders and the stay of mentally ill offenders of unsound mind in detention for minor offenses also showed a clear disadvantage for the patient group, who were admitted for an average of 4–5 years (Table 2).

**Table 1.** Relationship Between the Length of Stay in Detention and the Sentencing Range Provided for in the Criminal Code (StGB)

		Relation to the penalty range		
Offense	Penalty range	under	in the	about
Dangerous threat: § 107(2)	1–3 y	0	28.6%	71.4%
Serious coercion: § 106	6 mo to 5 y	0	63.6%	36.4%
Serious bodily injury: § 84	1–5 y	0	51.3%	48.7%
Murder/attempted murder: § 75	10 y to life	76.7%	23.3%	–
Sexual offenses: §§ 205–207	6 mo to 10 y	0	76.5%	23.5%
Robbery: § 131	6 mo to 5 y	0	42.9%	57.1%
Theft: §§ 127–129	6 mo to 5 y	0	40.0%	60.0%
Arson: § 169	1–10 y	0	62.5%	37.5%
Resistance to state power	6 mo to 5 y	0	100%	0

**Table 2.** Length of Stay in Penal Institutions and Detention Centers by Offense

Offense	Measure § 21/1 (*N* = 235)	Detention (*N* = 800)	*p*
Dangerous threat	4.2 ± 3.7	1.6 ± 2.9	0.049
Serious coercion	4.5 ± 2.8	2.6 ± 0.9	n.s.
Serious bodily injury	4.9 ± 4.9	2.5 ± 1.3	0.000
Murder/attempted murder	5.8 ± 6.4	11.2 ± 5.5	0.000
Sexual offenses	4.2 ± 5.6	3.9 ± 2.4	n.s.
Robbery	6.9 ± 8.4	4.4 ± 3.1	0.042
Theft	6.5 ± 3.1	2.2 ± 1.3	0.025
Arson	8.0 ± 4.8	5.5 ± 1.3	n.s.
Resistance to state power	2.1 ± 1.2	1.5 ± 2.6	n.s.

### Changes due to the new legislation

In all relevant legal texts, the term “institution for mentally abnormal offenders” has been generally changed to “Forensic Therapeutic Center,” which is intended to emphasize the therapeutic nature of this kind of detention. It is expected that this will also make more therapeutic resources available in the future. However, the more important changes concern the modalities of admission to detention and the responsibility for the treatment of persons provisionally admitted to detention.

By increasing the sentencing range to offenses punishable by more than 3 years, the legislature hopes to reduce the burden on the prison system. In addition, the aim is to prevent patients with minor offenses (resistance to state authority, dangerous threats) from remaining in detention for significantly longer than healthy offenders in prison for comparable offenses.

### Criminal placement in a forensic therapeutic center

*Any person who has committed an offense under the significant influence of a serious and persistent mental disorder and who cannot be punished solely because of being mentally incompetent (Section 11) at the time of the offense due to this disorder shall be placed in a forensic therapeutic center if there is a high probability that he/she will otherwise commit a punishable offense with serious consequences in the foreseeable future under the significant influence of his/her mental disorder. If there is such a fear, a person who, without being mentally incompetent, has committed an act pursuant to subsection (3) under the significant influence of a serious and persistent mental disorder shall also be placed in a forensic therapeutic center. In this case, placement shall be ordered at the same time as the sentence is imposed. Only acts punishable by more than 1 year’s imprisonment may give rise to a criminal detention order. If the threatened custodial sentence for this offense does not exceed 3 years, the apprehension under subsection (1) must relate to an offense of bodily harm punishable by more than 2 years’ imprisonment or to an offense against sexual integrity and self-determination punishable by more than 1 year’s imprisonment. Acts against another person’s property that are punishable by a custodial sentence are not considered to be a triggering offense unless they were committed using violence against a person or under threat of a current danger to the victims life.*

### Comment

Whereas under the old legislation, mentally ill or disturbed offenders who had committed an offense punishable by at least 1 year’s imprisonment could be committed, the de facto sentencing range has now been raised to 3 years. Only if there is a high probability of a repeat offense using bodily force that is punishable by more than 2 years’ imprisonment or if there is a high probability of acts against sexual integrity and self-determination that are punishable by more than 1 year’s imprisonment will the offender be committed to a detention facility.

On the one hand, the legislature obviously hopes that this will relieve the burden on the facilities by reducing the incidence of admissions, but on the other hand, the principle of proportionality will be upheld. However, the increase in the sentencing range creates a “blind zone.” Mentally ill persons who are mentally incompetent and have committed crimes punishable by 1–2 years, in particular patients who have made dangerous threats, have no further legal sanctions or conditions to fear. As an Austrian study has shown,^2^ civil law restrictions on liberty under the Hospitalization Act often fall short, especially in the case of patients who have been admitted due to dangerous threats. This group of people, in particular, was admitted under the Hospitalization Act much more frequently in the run-up to the crime, than, for example, sane patients who have committed a homicide. They frequently discontinued treatment in psychiatric wards, even under conditions of detention, fled the ward and were often no longer able to be readmitted. The Austrian Hospitalization Act recognizes 3 criteria for hospitalization that must be present at the same time: a. an acute psychiatric illness or disorder, b. an associated serious and significant danger to self or others, or c. the absence of an effective treatment alternative. If only 1 of these 3 criteria relevant to placement is not met, the placement must be lifted. As the willingness of mentally ill persons who pose a threat to others to undergo treatment is usually considered to be very low, the Hospitalization Act is likely to fall short of providing adequate treatment under the conditions of general psychiatric care for persons who have been committed to detention for making dangerous threats.

## Crisis intervention

*According to Section 157 of penal law, instead of revocation, the court shall suspend the provisional suspension of enforcement (Section 157 a) for a maximum period of 3 months and provisionally enforce the criminal placement if it can be assumed that treatment and care in a forensic-therapeutic center, a public psychiatric hospital, or a public hospital with a psychiatric ward can improve the condition of the person concerned during this period to such an extent that a continuation of the provisional suspension of enforcement is possible again.*

As inpatient detention facilities are generally operating at more than full capacity, it is to be expected that the courts will make frequent use of this option.

## Comment

This immediately led to a statement by the Austrian Society for Psychiatry and Psychotherapy (ÖGPP). As the currently valid and available planning principles of the healthcare system (Austrian Healthcare Structure Plan, Regional Healthcare Structure Plans) take into account the care needs of a region, but not the psychiatric care of offenders in terms of the execution of measures, this group of people is neither included in the existing structures nor in the current plans. Experience has shown that a longer planning and implementation phase will be required before the existing structures can be expanded appropriately. As the admission of offenders in the sense of the execution of measures can be imposed on psychiatric hospitals or psychiatric departments of public hospitals, it must be assumed that in this case, the spatial and personnel structures in the general psychiatric departments for other mentally ill persons will not be available to a sufficient extent.

For around 2 decades, the duration of inpatient treatment in psychiatric hospitals and psychiatric departments in general hospitals has mostly been reduced to a few days to a few weeks at most. Under the new law, in addition to 50–70 general psychiatric patients who can be discharged after 2–3 weeks, there would be 2 or 3 patients who would have to be treated for up to 2 years. It is doubtful that adequate treatment for forensic patients can be offered under these conditions.

It is considered extremely problematic to treat forensic patients together with general psychiatric patients. The different length of stay, the different risk prognosis, and the different legal and assessment practices alone must cause tensions between the 2 patient groups. This results in a considerable additional workload for the staff working in these areas. The shortage of nursing staff that has arisen in recent years is also increasingly noticeable in psychiatric hospital departments. An additional burden caused by the admission of forensic patients to general psychiatric wards is likely to encourage nurses to leave psychiatry.

Forensic psychiatry has developed considerably in recent years and is a highly specialized field within psychiatry with elaborate methods of prognosis and treatment. Forensic psychotherapy and criminal therapy, in particular, require a high level of expertise that is not available in general psychiatry. It would require time-consuming and intensive training for all professional groups working in this field in order to further develop the relevant knowledge, skills and abilities so that the dangerousness of forensic patients can be correctly assessed and treatment can be provided to the required quality.

The responsibility for securing, detaining and monitoring patients to be treated in general psychiatric wards as part of provisional placement is completely unclear. Within the existing structures, these requirements go beyond the given framework and pose a considerable risk potential both for the general psychiatric patients undergoing joint treatment and for the staff.

In recent decades, the former large psychiatric hospitals have largely been replaced by regional psychiatric departments at general hospitals. In addition to psychiatric departments, these general hospitals also have departments for internal medicine, obstetrics, pediatrics, and other medical specialties. As the psychiatric wards at general hospitals are often not locked, but are run openly, this means that their patients can sometimes leave the psychiatric ward without permission. If offenders are also admitted to these psychiatric wards without the staff being appropriately qualified, this also increases the risk for patients in other hospital departments.

### Involuntary treatment in the forensic system

Psychopharmacological treatment against the will of the person concerned is only possible in forensic psychiatry to avert a significant and immediate danger to themselves or others if no less severe means appear sufficient and promising for this purpose. The need for such a measure is determined by the attending physician. Authorization to carry out such measures is granted following a written application to the Ministry of Justice. Subsequently, in order to enable an external review, the doctor responsible must send a protocol of the procedure to the authorizing body.

This procedure is modeled on the procedure in general psychiatry in terms of documentation and indication.

## Relapse prevention

The treatment of the individual in correctional facilities, with all its difficulties and challenges, aims to reduce the specific danger posed by the illness to such an extent that reintegration into society is possible. This goal is pursued with a high expenditure of resources and always requires an individualized treatment of risk factors, the strengthening of resources, and the development of protective factors. Progress can be assessed in the course of gradual relaxation measures.

The success of this system can ultimately be measured, from a legal perspective, by the recidivism rates. And here, a long-term comparison consistently shows that patients who are released from the measure have a significantly lower reconviction rate than is the case for offenders with the same offenses.

## References

[1] Eher R, Domany S, Engel F. Monitoring report 2021 enforcement of measures pursuant to Section 21 (1) StGB; 2022.

[2] Meuschke N. Typological description of the inpatient utilization behavior of mentally abnormal lawbreakers suffering from schizophrenia. Unpublished diploma thesis; 2014.

[3] Stompe T, Keckeis K. Diagnosen, Delikte und Migrationshintergrund. *Clinicum Neuropsy*, 2017.

